# Automated Solid-Phase Subcloning Based on Beads Brought into Proximity by Magnetic Force

**DOI:** 10.1371/journal.pone.0037429

**Published:** 2012-05-18

**Authors:** Elton P. Hudson, Andrej Nikoshkov, Mathias Uhlen, Johan Rockberg

**Affiliations:** 1 School of Biotechnology, KTH – Royal Institute of Technology, Stockholm, Sweden; 2 Science for Life Laboratory, KTH – Royal Institute of Technology, Stockholm, Sweden; 3 The Novo Nordisk Foundation Center for Biosustainability, Technical University of Denmark, Hørsholm, Denmark; Deutsches Krebsforschungszentrum, Germany

## Abstract

In the fields of proteomics, metabolic engineering and synthetic biology there is a need for high-throughput and reliable cloning methods to facilitate construction of expression vectors and genetic pathways. Here, we describe a new approach for solid-phase cloning in which both the vector and the gene are immobilized to separate paramagnetic beads and brought into proximity by magnetic force. Ligation events were directly evaluated using fluorescent-based microscopy and flow cytometry. The highest ligation efficiencies were obtained when gene- and vector-coated beads were brought into close contact by application of a magnet during the ligation step. An automated procedure was developed using a laboratory workstation to transfer genes into various expression vectors and more than 95% correct clones were obtained in a number of various applications. The method presented here is suitable for efficient subcloning in an automated manner to rapidly generate a large number of gene constructs in various vectors intended for high throughput applications.

## Introduction

Versatile, reliable, and fast DNA cloning platforms are essential to the creation of novel synthetic biological systems, in which it is often desirable to interchange genes and other functional elements within a vector or between vectors. A range of subcloning methodologies has been developed for the synthesis and manipulation of genetic constructs, each with an unique dependence on endonuclease [1], [2], recombinase [3], [4], or exonuclease [5], [6] treatment prior to insert-vector annealing and ligation. Several methods eliminate the need for restriction enzymes and rely solely on polymerase extension to create gene constructs [7], [8]. Recently the 16.5 kbp mouse mitochondrial genome was synthesized *in vitro* from overlapping oligos [9]. These solution phase methods are powerful and versatile, but can be hampered by the incompatibility of required enzymes, time-consuming DNA purifications, or intermediate bacterial transformation to repair and amplify intermediate constructs.

The immobilization and hybridization of DNA onto paramagnetic beads or other solid-phases have shown to be invaluable in several applications including next generation DNA sequencing [10], [11], genotype/phenotype coupling [12]–[14], microfluidics-based biosensors [15] and expression-profiling chips [16]. A solid-phase bead platform, in which one or multiple DNA components are attached to a solid support, would provide a physical handle on the DNA construct, allowing for quick purification eliminating the need for gel or spin column purification steps, but also providing easy analysis, using flow-based sorting at any step in the cloning process [10]. Such a cloning platform could lead to reduced side reactions, lower background and enable high throughput automation.

Here we show a subcloning platform in which target genes are inserted into bead-immobilized acceptor vectors via solid-phase annealing and ligation. To demonstrate the utility of the platform, we have cloned human gene fragments, coding for potential cancer therapeutic targets, into bacterial, fungal, and mammalian expression vectors. We also demonstrate scale-up and automation of the platform and use it to transfer 95 target genes from a donor vector into a mammalian expression vector.

## Results

### The principle of the solid phase cloning method

**Figure 1:**
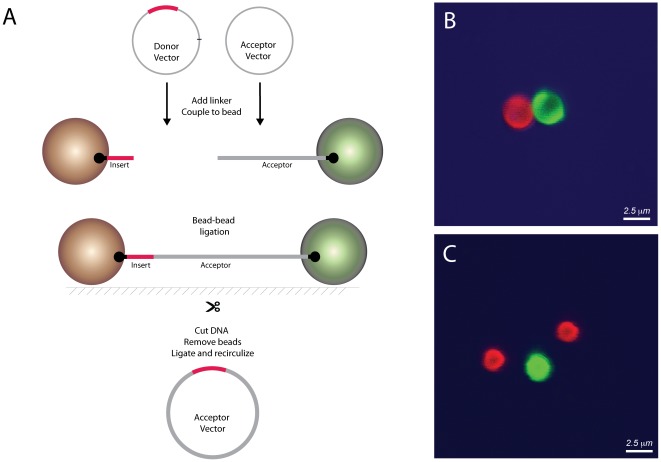
Bead-based subcloning strategy. (**A**) An insert is excised from its donor vector, and capped with a biotin-containing oligo in a one-pot reaction. The insert is then attached to a streptavidin-coated bead and ligated to an immobilized acceptor vector, which has a compatible restriction site (here NotI). A non-productive fragment of the donor vector is also attached to bead, but cannot participate in further ligation steps due to a non-compatible restriction site (AsiSI). The DNA construct is released from bead via cutting at unique restriction site (here AscI). Details are found in Supplemental Information. (**B**) Confocal microscope image of an inter-bead ligation. Acceptor-vector beads are green (Alexa647 label) and insert beads are red (Alexa488 label). The insert is ITGA2b (1 350 bp) and the acceptor vector is pLentiHAp (8 180 bp). The inter-bead distance is much shorter than that expected for an extended DNA ligation product (ligation product expected at 9 530 bp, or 3.2 µm), which suggests multiple inter-bead connections. (**C**) A three-bead ligation. The target gene and acceptor vector are the same as in (A) Here the interbead distances are larger, indicating fewer DNA connections between the beads.

A schematic view of the solid-phase cloning platform is shown in Figure 1A. The solid-phase cloning platform is centered upon an immobilized acceptor vector, which is linearized, biotinylated at one end and attached to streptavidin-coated paramagnetic beads [17]. The free end is modified to allow for subsequent DNA addition (e.g. treated with a restriction endonuclease). Near the biotin-streptavidin linkage is a unique restriction site (here AscI), which is used to release the vector from the bead. The target genes are inserted into bead-immobilized acceptor vectors via solid-phase annealing and ligation. Typical bead loadings were approximately 1500 DNA molecules per bead (0.5 ng vector DNA/µg bead for a 5,000 base pair vector). The target gene can be in the solution phase, as a PCR product or excised from a donor vector, or can itself be attached to a bead. After target-vector ligation, the beads are collected via magnet and excess reagents are washed away. The beads can subsequently be used in additional reactions or the construct can be removed from the bead for transformation. The beads are large enough to be detected by fluorescent activated cell sorting (FACS). This facilitated optimization of the ligation protocols with fluorescent-labeled DNA.

We constructed a panel of solid-phase acceptor vectors that can be used to express genes in several hosts (see Supplemental Information Table S1 for vector details). The vector beads can be prepared in bulk and stored at 4°C for several months or −20°C for one year without loss of reactivity (data not shown).

### DNA addition to immobilized vector from solution phase

**Figure 2:**
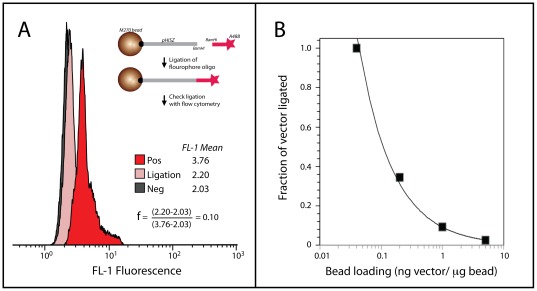
Ligation of solution phase DNA to a bead-immobilized vector. (**A**) A fluorescence-based assay for determining extent of ligation. Beads with immobilized vector are incubated with a Alexa 488 fluorescent oligo. The extent of ligation is measured via flow cytometry. Bead loading was at 1 ng vector DNA (pHISZ)/ug bead vector. Positive: Beads in which pHISZ vector is fully fluorescently labeled. Ligation: Beads after ligation. Negative: Beads in which pHISZ vector is not fluorescently labeled. The extent of ligation f is measured as a percentage of the Positive signal. (**B**) Extent of ligation is reduced at high bead loadings.

Initial tests focused on the capture of a solution-phase fluorescently labeled oligonucleotide to an immobilized acceptor vector in a solution-to-solid ligation (see schematic in Figure 2A) allowing a fluorescence-based assay to measure ligation efficiency. The extent of ligation was measured and compared with the fluorescence intensity to that of a positive control, in which all acceptor-vector DNA was fluorescent. A representative comparison is shown in Figure 2A. A dramatic dependence of ligation extent on bead loading was observed, with 100% ligation observed at the lowest bead loading (Figure 2B). Importantly, vigorous stirring and the addition of 5% vol/vol PEG 4000 were required for appreciable ligation. The extent of ligation extent measured via cytometry correlated with ligation efficiency as colony forming units (cfu) per ng vector when the vector was cut from the bead and circularized (data not shown).

To test the robustness of the solution-to-solid ligation, gene inserts of different sizes were ligated to the vector and used for bacterial transformation. Three DNA inserts (500 bp–3000 bp) were excised from the donor vector and ligated to two separate, immobilized acceptor vectors, the *Pichia pastoris* expression vector and the *Staphylococcus carnosus* expression vector. After ligation the constructs were released from beads via AscI treatment, circularized, and used for *E. coli* transformation (Table 1). Alternatively, DNA constructs can be eluted from beads via heat treatment ([18]).

**Table 1:** Ligation efficiencies of bead-based subcloning strategies.*

Acceptor Vector	Size (kb)	Donor Insert	Size (kb)	Solution-Bead		Bead-BeadMAGNET ON		Bead-BeadMAGNET OFF	
				Correct	cfu/ng	Correct	cfu/ng	Correct	cfu/ng
pPICZαCp	3.6	TNF	0.47	5/8	60	7/8	550	7/8	100
		EGFR	1.86	7/8	30	8/8	880	4/7	300
		ITGAL	3.19	7/8	30	7/8	750	4/8	350
pSCEM2	7.6	TNF	0.47	7/8	50	13/13	750	2/8	90
		EGFR	1.86	7/8	70	12/13	1500	5/8	610
		ITGAL	3.19	8/8	50	12/13	820	6/12	340

* *Vector and donor beads were loaded at 0.5 ng DNA/µg bead.*

The solution-to-solid phase subcloning gave ligation efficiencies (∼50 cfu/ng vector DNA) much lower than a fully solution-based protocol (typically ∼1 000 cfu/ng; cells were competent at 10 000 cfu/ng pUC19). Notably, ligation efficiencies were not dependent on gene insert or vector size (Table 1). We also tested the ability to attach inserts as PCR products, in which the NotI and AscI sites were added via primers. PCR amplicons were treated with NotI-AscI and phosphatase before ligation to immobilized acceptor vector. They were not purified from the PCR reaction mixture. This approach gave lower (∼20–30 cfu/ng) ligation efficiencies.

### Bead-bead mediated sub-cloning

We hypothesized that ligation efficiency at higher bead loadings could be improved if both vector and gene DNA were immobilized onto beads; here the paramagnetic beads could be brought into very close contact with a magnet. This bead-bead subcloning process is depicted in Figure 1A. The gene is excised from its donor vector in a series of one-pot enzymatic reactions, capped with a biotinylated oligo, and attached to bead (see Supplemental Methods S1 and Supplemental Figures S1, S2, S3 for details). A second DNA fragment is also attached to bead, but its non-complementary restriction site prevents it from participating in subsequent ligation steps. Excess reagents and non-biotinylated DNA are then removed. The donor- and acceptor beads are then co-incubated with DNA ligase in the presence of a magnet. Alternatively, the insert DNA can be prepared via PCR with a biotinylated primer. In this way it can be attached to the bead, treated with NotI, and ligated to vector beads.

We sought to characterize the critical bead-bead ligation step using both confocal microscopy and flow cytometry. We attached red and green fluorophores to gene- and vector beads, respectively. After ligation, the beads were examined qualitatively with a confocal microscope or quantitatively using a flow cytometer.

Representative microscopy images are shown in Figures 1B and 1C. Figure 1B shows a pairwise bead-bead construct and Figure 1C shows a rare three-bead construct in which a single acceptor bead is attached to two insert beads. These multi-bead constructs migrated together on the slide surface, indicating that the beads were attached through their DNA. The bead-bead constructs were non-uniform with regard to inter-bead distance, which suggests multiple DNA attachments between beads. The gene ITGAL2b is 1350 bp, corresponding to approximately 0.46 µm for extended B-form DNA [19], while the magnetic beads have a diameter of 2.8 µm. The acceptor vector pLentiHAp is 8180 bp (2.76 µm). Successful constructs would be expected at 9 530 bp (approximately 3.22 µm inter-bead distance). Constructs showing substantially shorter inter-bead distance (e.g. Fig. 1B) may be indicative of multiple DNA connections, while those showing the 3.22 µm theoretical distance for a ITGAL2b-pLentiHAp construct (e.g. Fig. 1C) suggest fewer DNA connections.

**Figure 3:**
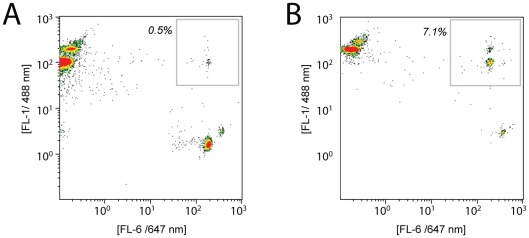
The presence of magnet increases the extent of ligation in bead-bead subcloning. Flow cytometry of fluorescent-labeled beads allows quantification of bead-bead ligations. Insert beads are red-labeled (Alexa488 label) and acceptor-vector beads are green-labeled (Alexa647 label). Successful bead-bead ligations appear at high FL-1 and FL-6 readouts (boxed). The insert is ITGA2b (1 350 bp) and the acceptor vector is pLenti1 (8 180 bp). (A) Ligation in the absence of magnet. Approximately 0.5% of acceptor beads are ligated. (B) Ligation in the presence of magnet. Approximately 7% of the acceptor beads are ligated, a 14-fold increase in extent of ligation.

Flow cytometry allowed quantification of the success of the bead-bead ligation reaction. Multi-bead constructs have a dual-color signature, as well as increased forward- and side-scatter. Figure 3A and 3B show typical cytometry plots in which a small population of dual-colored bead constructs are observed (gray boxes in 3A and 3B). The amount of acceptor beads ligated to insert ranged from 0.5% (no magnet during ligation) to 7.1% (magnet on during ligation). In all cases, >90% of acceptor beads were not ligated to donor beads.

### Bead-bead ligation is enhanced in presence of magnet

We expected gene-vector ligation efficiency to be greatly reduced in the solid phase reaction, partially due to the slow motion of the beads. To facilitate bead-bead contact we performed the ligation in the presence of a magnet, which brings the paramagnetic insert and acceptor-beads into close contact. To test the robustness of the procedure, we also investigated the effect of gene and vector length on ligation efficiency.

Table 1 shows the ligation efficiency for several gene-vector combinations spanning a range of DNA lengths in the presence and absence of magnet. Under optimal conditions (gene and vector on beads, magnet on), we obtained ligation efficiencies of ca. 800 cfu/ng acceptor DNA, which is similar to that obtained for solution–phase cloning methods such as Gateway (ca. 2000 cfu/ng [3]).

The ligation efficiency and accuracy were not dependent on the length of the gene or vector DNA. The presence of magnet had a strong positive effect on ligation efficiency. For example, in the ligation of TNF to the *S. carnosus* surface display vector pSCEM2, the ligation efficiency was reduced 8-fold when the magnet was not used during ligation. We also observed a reduced number of successful clones, indicating vector re-ligation. The reduction in ligation efficiency in the absence of magnet is consistent with the change in the number of bead-bead constructs observed via flow cytometry.

Notably, bead-bead ligation efficiency was reduced at higher DNA loadings. Increasing the vector DNA loading from 0.5 ng to 2 ng DNA/µg bead reduced the pSCEM2-TNF and pSCEM2-EGFR bead-bead ligation efficiencies by 2.4 fold and 1.8 fold, respectively. This is consistent with the observed decrease in ligation with bead loading in the solution-to-solid ligations (Figure 1B).

### Automation of the solid-phase subcloning platform

We envisioned using the solid-phase cloning platform to rapidly switch gene cassettes between vectors in a large-scale manner. We programmed the optimized bead-bead protocol to a Magnatrix 8000 robotic liquids handler and used it to transfer 95 genes from the vector pAff8c to the mammalian expression vector pLentiHAp in a 96-well format. The genes ranged in size from 90 bp to 3300 bp (details of the genes and construction of the pAff8c library can be found in Supplemental Methods S1 and Table S3). Reagents were combined into stock solutions to minimize pipetting time, and beads were washed using magnetic separation. Completed vectors were used to transform *E.coli* in a 96-well format. The total subcloning process, excluding transformation, was completed in approximately 3 h.

**Figure 4:**
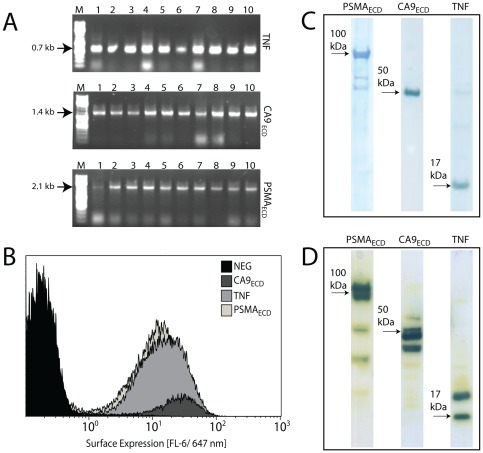
Bead-bead subcloning of cancer genes into multiple expression vectors. The ectodomains of three cancer antigens (TNF, CA9 ectodomain, and PSMA ectodomain) were subcloned via bead-bead subcloning from an *E. coli* expression vector into expression vectors for *S. carnosus, P. pastoris*, and CHO. (A) PCR screens of pLentiHAp transformants show correct sizes for 10/10. (B) Expression of target proteins on the surface of *S. carnosus* using the surface display vector pSCEM2. The surface expression is quantified via flow cytometry as HSA-Alexa647 binding to ABP, which is co-expressed with the target protein. (C) Expression of target proteins from the CHO expression vector pLenitHAp. Western blotting (anti His6) of CHO cell lysates. (D) Expression of target proteins from the *P. pastoris* expression vector pPICZap. Western blotting (anti His6) of *P. pastoris* cell lysates.

The average ligation efficiency for the automated procedure was 200 cfu/ng and not effected by gene size. We PCR-screened at least 6 colonies from each plate and observed >95% correct inserts, which was also independent of gene size (Table 2). All plates had at least one clone with desired insert. Figure 4A shows the 100% ligation accuracy observed for three representative genes, TNF, CA9 ectodomain, and PSMA ectodomain.

**Table 2:** Statistics of automated bead-bead subcloning of 95 target genes.

Sampling	Gene Size (bp)	Number of Inserts	Correct Insert (%)
1	500>x	39	98
2	1000>x>500	29	93
3	2000>x>1000	16	98
4	x>2000	11	94
5	3200>x>100	95	96

### Parallel cloning and protein expression from completed vectors

One application of the solid-phase subcloning platform is the rapid construction of vectors for the expression of difficult proteins in multiple hosts, for functional analysis or for use in screening platforms. For example, expression of human proteins in *E. coli* often leads to misfolding and low expression of soluble protein [20]. It is often necessary to produce these proteins in fungal or mammalian hosts.

To demonstrate the potential of the solid-phase subcloning platform in aiding protein-expression studies, we chose three genes from our collection of clinically relevant genes (TNF, CA9 ectodomain and PSMA ectodomain) and subcloned them from the donor vector pAff8c into three expression vectors (pSCEM2, pPICZαCp and pLentiHAp). DNA sequencing of successful clones verified correct in-frame ligation of the target gene into the expression vector. Of the three genes, only TNF was successfully expressed in *E. coli* (data not shown).

Figure 4B shows the *S. carnosus* surface-expression of the target genes from the pSCEM2 vector as determined by flow cytometry. TNF, CA9 ectodomain and PSMA ectodomain are expressed (increased FL-6 fluorescence, for details of this assay see Materials and Methods). These clones can be used in binding assays for therapeutic antibodies. A similar expression pattern was observed in mammalian cell culture (CHO; Fig. 4C), with each protein appearing at its expected molecular weight. Each protein was also produced the fungal host *P. pastoris* (Fig. 4D), although the presence of several bands around the expected molecular weight may indicate an alternative glycosylation pattern from this host [21]. Additionally, the CA9 protein was secreted at high titers into the CHO culture medium, while secretion was hindered in the *P. pastoris* cultures (data not shown). Solid-phase vector construction could be used in tandem with a high-throughput protein expression and purification platform, such as one recently developed in our laboratory [22].

## Discussion

We have developed a subcloning platform based upon the immobilization of DNA onto paramagnetic beads. The protocol can be used with PCR products or, in cases where additional PCR is not desirable, with inserts excised from donor vectors. We have circularized DNA after elution from beads for transformation into *E. coli*. However, linear DNA could be used directly in transformations of hosts capable of linear-DNA uptake. Alternatively, whole bead-DNA constructs could be used for transfections [23].

The introduction of the solid phase presents unique problems compared to solution-phase reactions, such as enzyme-to-bead mass-transfer limitations [24] and potentially nonproductive conformations of DNA on the solid surface [25]. We observed higher ligation efficiencies with lower bead surface coverage, consistent with previous studies of DNA hybridization onto flat surfaces [26] and lowest ligation efficiencies from solution-to-bead ligations. These efficiencies were nearly an order of magnitude lower than those of solution-phase ligations. This discrepancy is most likely due to mass-transfer limitations present in the heterogeneous ligation reaction, as the ligation efficiencies were increased dramatically when the mixture was stirred. It may also be due to the inaccessibility of the vector DNA on the bead surface, as the addition of PEG increased ligation efficiencies. PEG is known to facilitate the unfolding of DNA structures [27]. Additionally, *intra*-bead bridging between proximal vector strands via ligation of compatible sticky-ends may hinder addition of the gene insert. This problem would be exacerbated at high vector densities, which is consistent with the observed reduced ligation efficiencies at high bead loading (Figure 2B). While we have taken measures to prevent *intra*-bead bridging via extensive dephopsphorylation of vector ends, the problem could be eliminated completely by adaption of other cloning methods, which do not rely on endonucleases, such as exonuclease “chew-back and anneal” methods [6]. We expect such methods to be compatible with the solid-phase platform.

Solid-to-solid ligations showed higher efficiencies than the solution-to-solid ligations. This may be due to a proximity effect; once two beads are initially linked to one another through DNA strands, the attachment of subsequent DNA strands between them is facilitated as in an intra-molecular reaction. Such proximity effects are not present in the solution-to-solid ligations. This hypothesis is supported by the nearness of some bead-bead constructs as seen via in confocal microscopy. The presence of the magnet increased the ligation efficiency still further, due to an increased propensity to form an initial bead-bead ligation. This was confirmed by a large increase in the number of linked beads observed by flow cytometry when compared to ligations without magnet. These results suggest that it would be possible to control the extent of ligation quite easily by judicious application of a magnet.

The solid-phase ligation efficiencies were independent of insert and vector sizes in the range tested (90–3300 bp). This is consistent with previous solid-phase DNA manipulation studies, which demonstrated both efficient enzyme cleavage and strand DNA hybridization at sites >20 nt from the biotin immobilization site [28]. The ease of constructing vectors >10000 bp (e.g. ITGA2B-pLentiHAp) suggests that longer dsDNA constructs are possible given the correct buffer conditions. We did not observe a dependence of ligation efficiency on GC content of either vector or insert. We expect that the use of dsDNA reduces the risk of secondary structures such as hairpins, which may hinder ligation. Secondary structure may be necessary to consider if adapting the method to ssDNA annealing [25].

The ability to quickly pull out bead constructs via magnetic separation eliminated the need for gel extractions and spin-columns and simplified automation of the protocol. The sublconing process could be fully automated, with bead capture, enzymatic digestion, and DNA washing performed by a robot. Solid-phase cloning could be adapted to recently-developed microfluidics devices which are designed to manipluate magnetic microparticles with high precision [29]. Such a platform could be used for combinatorial vector construction on a scale not possible with solution-phase cloning.

An automated platform would also greatly aid high-throughput expression and function studies. We envisioned a scenario in which several expression hosts would be tested for the optimal expression of a panel of proteins. Using our optimized protocol, in which restriction digests were performed simultaneously and reagents were combined and stored as stock solutions, we were able to transfer 95 target genes from a donor vector to a mammalian expression vector in 3 h. A preliminary test of several of these proteins in different expression vectors showed interesting discrepancies in terms of secreted titer and glycosylation state.

An additional direction of the bead-based cloning method is assembly of multicomponent constructs via sequential addition of genetic elements [30]. The method could aid synthetic biology efforts by accelerating the construction of vectors via the BioBrick [1], BglBrick [2], and USER [31] methods by eliminating intermediate transformation steps. The ease of purification of cloning intermediates could potentially ease assembly of long constructs. The solid-phase platform could also be easily extended and adapted for other DNA manipulation strategies, such as ligase-independent and endonuclease-free methods.

In conclusion, the approach shown here has demonstrated the power of magnetic beads for standardized highly automated subcloning of large number of fragments into expression vectors for protein expression screening and we point out a direction for use of magnetic beads in broader terms for generation of longer multicomponent genetic assemblies, relevant for metabolic engineering.

## Methods

All enzymes were from Fermentas UAB (Vilinius, Lithuania) and were FastDigest. We found the use of these FastDigest enzymes to be critical for simultaneous restriction digestions.

Genes for *solution-to-bead* and *bead-to-bead* ligations were from an *E. coli* “donor” vector pAff8c [32]. We created a library of 95 pAff8c vectors encoding the full and partial ectodomains of 65 cancer antigens (for cloning procedures and antigen details see Supplemental Methods s1).

Acceptor beads were prepared in bulk and used in solution-to-solid and solid-to-solid ligations. Briefly, acceptor plasmids were linearized (NotI/AscI) and a biotinylated linker containing a complementary AscI restriction site was attached to one end. The plasmids were then incubated with DynaBeads M270 streptavidin-coated beads (Invitrogen, Carlsbad, CA) to create acceptor beads. Typical bead loadings were 0.5 ng plasmid DNA/µg beads, corresponding to ca. 1 500 DNA/bead. Details on acceptor vectors, including primer sequences, see Supplemental Information Table S1, S2, S3.

### Solution-to-bead ligations

Vector beads were first treated with NotI (37 C 1 hr), which produced a free NotI end, and then washed twice in Tris buffer pH 8. Genes from the pAff8c clone library were prepared as NotI/AscI-digested, purified fragments, or as NotI/AscI-digested PCR products. The ligation reaction volume was 50 µL and included 5 µg of acceptor beads (loaded at 0.5 ng DNA/µg beads), a 10∶1 molar excess of insert DNA, 5 µL of T4 DNA ligase buffer, 5% v/v PEG4000 and 1 µL T4 DNA ligase. The ligation reaction proceeded at room temperature for 45 min under shaking. The beads were removed via magnetic separation and washed. The DNA was removed from beads with AscI treatment (30 µL, 37°C 45 min with vigorous shaking). The new vector constructs were circularized with addition of T4 DNA ligase and 0.5 mM ATP (22°C 10 min) and 3 µL used for bacterial transformation.

Solution-to-bead ligation of the fluorescent capping oligo to M270-pHISZ was performed similarly but used 10 pmol of an 18 bp oligo containing a BamHI site and a 5′-AlexaFluor 488 (Eurofins MWG Operon, Ebersberg, Germany). For the positive control, the pHISZ vector was capped with the BamHI oligo before bead loading, ensuring near 100% ligation and maximal fluorescence at each loading.

### Bead-to-bead ligations

Solid-to-solid bead ligations were performed with acceptor beads and donor beads. Donor beads were prepared by incubating streptavidin-coated M270 beads with biotinylated genes from the pAff8c clone library. Biotinylated genes were either from PCR with biotinylated primers or constructed via excision from the pAff8c vector followed by addition of a biotinylated oligo. For details of the preparation of donor beads see SI Methods.

We performed the solid-to-solid ligation of acceptor and donor beads in 1.5 mL eppendorf tubes. For ligations in the presence of magnet we used a magnetic separation stand. In each case the ligation volume was 50 µL, included 1 µg of M270-acceptor beads (∼0.5 ng acceptor plasmid) and 10 µg of M270-donor beads (∼5 ng donor DNA), 5 µL of T4 DNA ligase buffer, 5% v/v PEG4000 and 1 µL T4 DNA ligase. The ligation reaction proceeded for 60 min, with pipette stirring every 10 min. DNA was removed from beads with AscI treatment, circularized and used for transformation as described above.

### Flow Cytometry and Confocal Microscopy

Fluorophores AlexaFluor 488 and AlexaFluor 647 succinimidyl esters were from Invitrogen. Acceptor and donor-loaded beads were modified at pH 9 with AlexaFluor 647 and AlexaFluor 488 respectively, according to the supplier's protocol (50 µg donor beads and 50 µg acceptor beads were incubated with 50 µg of fluorophore for 1 h at 22°C). In this reaction the streptavidin is covalently modified at its amine groups. The beads were washed 3 times and re-suspended in Tris buffer pH 8. The donor-beads were loaded at 0.5 ng DNA/µg bead with ITGAL2b cut (NotI AscI) from the vector pAff8c and the vector beads were loaded with pLentiHAp at 0.5 ng DNA/µg bead. Donor-acceptor ligations were performed with the labeled beads according to the above protocols (1 µg acceptor beads, 10 µg donor beads, 30 µL reaction) in the presence and absence of magnet.

The ligation mixture was analyzed with a Beckman-Coulter Gallios flow cytometer using FL-6 (647 nm) and FL-1 (488 nm) excitation and detection. Before analysis the ligation mixture was diluted to 300 µL in Tris buffer pH 8. Fifty thousand events were recorded for each sample.

### Automation of the subcloning procedure

The subcloning protocol was programmed onto the Magnatrix 8000 robot (NordDiag A/S, Oslo, Norway) using the manufacturer's software. The pAff8c-donor library was used in 96-well format. Enzymes, M270 beads and acceptor beads were prepared as reagents and stored at 4°C during the procedure. Details of the protocol can be found in Supplemental Methods S1.

### Protein expression

The target proteins TNF, CA9 and PSMA were expressed in *E. coli, P. pastoris, S. carnosus* and Chinese Hamster Ovary Cells (LGC-labstandards AB, Borås, Sweden).

*E. coli RR1ΔM15* was transformed with pAff8c-target vector by heat shock and subjected to Km selection. Colonies were PCR screened with primers N2 and U5 and sequenced verified. Picked colonies were used to inoculate 10 mL of TSB medium (+Km) and grown overnight at 37°C. A culture aliquot (100 µL) was used to inoculate 50 mL of TSB (+Km) which grew to OD600 = 0.8 prior to induction with 0.5 mM IPTG. The cultures were moved to 25°C. After 6 hours the cells were pelleted, lysed with 5 mL of 7 M GdHCl (37°C 3 h). Lysis supernatant (5 µL) was run on SDS-PAGE gel.

*Staphylococcus carnosus* was transformed with pSCEM-target vector via electroporation according to a published protocol [33] and subjected to Cm selection. Colonies were PCR screened with primers SAPA23 and SAPA24. Picked colonies were used to inoculate 10 mL of TSB medium (+Cm) and grown overnight at 37°C. The pSCEM2 vector encodes a surface-anchoring scaffold upstream of the MCS. This scaffold includes albumin-binding protein (ABP). The successful expression of the scaffold+target protein is detected by incubation of transformed cells with fluorescent-labeled HSA. Cells were analyzed via flow cytometry (Beckman Coultier Galios) with FL-6 (647 nm) excitation.

*Pichia pastoris* strain SMD1168H and the *P. pastoris* expression vector pPICZα-C (Invitrogen) were a kind gift from Dr. Harry Brumer. *P. pastoris* was transformed with 0.5 µg pPICZαCp-target vector via electroporation according to the protocol of Wu [34], which was followed exactly except that the vector DNA was linearized with *PmeI* before electroporation. Transformation mixtures were subjected to Zeomycin selection (125 µg/µL) and grown at 30°C. Colonies appeared after ca. 3 days. Colonies were lysed with Lyticase (Sigma) and PCR-screened [35] with both AOX and target-specific primers and sequence verified.

Successfully transformed *Pichia pastoris* colonies were cultivated in 50 mL solutions BMGY and BMMY at 25°C according to the Invitrogen protocol, with daily addition of MeOH to 0.5% v/v. After 96 hrs the cells were pelleted and lysed. Lysis supernatants were analyzed via Western blot, with anti-His6 antibodies (Sigma) and HRP detection (Sigma).

Mammalian cells CHO-S (ATCC) were transfected via lentiviral delivery, as described in detail in Supplemental Information. Transfected cells were selected via puromycin resistance. Cells were cultured for one week after transfection at a maintained 70–80% confluence then harvested and sonicated. Lysates were filtered and analyzed via Western blot, with anti-His6 antibodies (Sigma-Aldrich, St. Louis, MO) and HRP detection (Sigma-Aldrich, St. Louis, MO).

## Supporting Information

Table S1 Vectors used in this study.
(DOC)

Table S2 Oligos used in this study.
(DOC)

Table S3 Antigens in the pAff8c donor library.
(DOC)

Figure S1 Detailed bead-based subcloning strategy.
(TIF)

Figure S2 Size distribution of pAff8c donor library.
(TIF)

Figure S3 Detailed schematic of donor-bead construction and characterization.
(TIF)

Methods S1 Preparation of acceptor plasmids. Preparation of acceptor beads from acceptor plasmids. Preparation of pAff8c library. Preparation of donor beads from pAff8c library. Automation of bead-to-bead protocol. Transfection of CHO-S and mammalian cell culture.
(DOC)
