# Supplementary material for: Automated Solid-Phase Subcloning Based on Beads Brought into Proximity by Magnetic Force
Source: PLoS One. 2012 May 18;7(5):e37429. doi: 10.1371/journal.pone.0037429 (PMC3356258; doi:10.1371/journal.pone.0037429)
Supplement: Table S1 — Vectors used in this study. (DOC) [file pone.0037429.s001.doc]

| **Table S1.** Vectors used in this study | | |
| --- | --- | --- |
| **Vector** | **Description** | **Size**  **(kb)** |
| pAff8c | *E. coli* intracellular vector. ”Donor” vector in bead-to-bead ligations | 5.6 |
| pHISZ | *E. coli* intracellular vector, used in fluorescent capping study | 2.8 |
| pSCEM2 | *S. carnosus* surface display vector | 7.8 |
| pPICZαCP | *P. pastoris* expression vector, modified from commercial pPICZαC | 3.8 |
| pLentiHAP | Mammalian expression vector, modified from commerical pLentiHAIII | 8.2 |
