# Supplementary material for: Automated Solid-Phase Subcloning Based on Beads Brought into Proximity by Magnetic Force
Source: PLoS One. 2012 May 18;7(5):e37429. doi: 10.1371/journal.pone.0037429 (PMC3356258; doi:10.1371/journal.pone.0037429)
Supplement: Table S2 — Oligos used in this study. (DOC) [file pone.0037429.s002.doc]

| **Table S2.** Oligos used in the construction of acceptor vectors and primers | | |
| --- | --- | --- |
| **Oligo** | **Description** | **Sequence** |
| LINK_F  LINK_R | Linking oligos. Contain AscI site and a 5’ biotin | 5’ CGCGCCTTCTGCAGGATCCTT 3’  3’ GGAAGACGTCCTAGGAA 5’ (biotin) |
| BamCAP_F  BamCAP_R | Fluorescent cap oligos for pHISZ. Contain BamHI site and a 5’AF488 | 5’GATCCACTGACTCGCATCCGATCGTG 3’  3’ GTGACTGAGCGTAGGCTAGCAC 5’ (AF488) |
| pPICZαC_ins | Insertion into EcoRI and XbaI of pPICZα-C to form pPICZα-CP. Contains NotI and AscI (italics). | 5’ GAATTCACATATGGGAAGCACGAGCTCCTCC*GCGGCCGC*TGCGAGC*GGCGCGCC*ACATCTAGA 3’ |
| pLenti_ins | Insertion into XmaI and XhoI of pLenti-HA to form pLenti-Hap. Contains NotI, AscI (italics), 6-His and Igk signal sequence (bold) | 5’ CCCGGGATGGAGACAGACACACTCCTGCT**ATGGGTACTGCTGCTCTGGGTTCCAGGTTCCACTGGTGAC**TCCGGGCATCACCATCACCATCACCATATGTCCATGAGCTCC*GCGGCCGC*TGGCTCT*GGCGCGCC*ACTCGAG 3’ |
| SAPA23 | pSCEM2 sequencing primer F | 5’ GGCTCCTAAAGAAAATACAACGGC 3’ |
| SAPA24 | pSCEM2 sequencing primer R | 5’ TGTTGAATTCTTTAAGGGCATCTGC 3’ |
| AOXF | pPICZαCp sequencing primer | 5’ GACTGGTTCCAATTGACAAGC 3’ |
| AOXR | pPICZαCp sequencing primer | 5’ GCAAATGGCATTCTGACATCCTCTTG 3’ |
| CMVF | pLentiHAp sequencing primer | 5’ CGCAAATGGGCGGTAGGCGTG 3’ |
| CMVR | pLentiHAp sequencing primer | 5’ TAGTCAGCCATGGGGCGGAGA 3’ |
