# Supplementary material for: Automated Solid-Phase Subcloning Based on Beads Brought into Proximity by Magnetic Force
Source: PLoS One. 2012 May 18;7(5):e37429. doi: 10.1371/journal.pone.0037429 (PMC3356258; doi:10.1371/journal.pone.0037429)
Supplement: Table S3 — Antigens in the pAff8c donor library. (DOC) [file pone.0037429.s003.doc]

| **Antigen** | **Length (bp)** | **Antigen** | **Length (bp)** |
| --- | --- | --- | --- |
| Metf | 3215 | IL12A | 597 |
| ITGAL_ECD_full | 3195 | CD2_ECD_full | 576 |
| MYO1A | 3126 | TREM-1 | 555 |
| ITGA2b_ECD_full | 2886 | ITGAL_ECD_I | 549 |
| ITGA4_ECD_full | 2832 | HGF_NK1 | 546 |
| KDR_ECD_full | 2232 | CD4_ECD_2D | 543 |
| TIE-1 | 2229 | EGFR_D3 | 519 |
| ADD3 | 2121 | EGFR_D1 | 501 |
| ITGB3_ECD_full | 2073 | TNFR_SF9 | 492 |
| FOLH1 | 2028 | MS4A1_full | 489 |
| AMOT_full | 2028 | IL1 | 477 |
| FLT1_ECD_full | 1974 | TNFa | 468 |
| HSP90 | 1911 | TNFR_SF10 | 468 |
| ERBB4 | 1878 | TNF_SF13 | 453 |
| ERBB3 | 1872 | EGFR_D2 | 447 |
| EGFR | 1863 | EGFR_D4 | 432 |
| ERBB2 | 1809 | IL2 | 396 |
| GAD2 | 1743 | IL17 | 387 |
| ITA2b_ECD_C | 1551 | IL4 | 387 |
| DLL4_C | 1485 | FOLH1_D3 | 384 |
| ANGPT2_full | 1440 | CLTA4_ECD | 384 |
| ITA2b_ECD_H | 1368 | IL9 | 378 |
| CSF2Rb | 1251 | IL13 | 360 |
| ITGB3_ECD_H | 1242 | NGF | 360 |
| CA9_ECD_full | 1134 | IL2Ra_DII | 354 |
| CD4_ECD_full | 1104 | VEGF | 351 |
| HSP90M | 1074 | CD33_ECD_V | 351 |
| IL5R_ECD_full | 948 | IL5 | 351 |
| FOLHD1 | 933 | CD40_ECD_S | 345 |
| IL12B | 909 | IL15 | 339 |
| CA9_ECD_CD | 897 | MSTN | 327 |
| CSF2Ra | 891 | CD2_ECD2_V | 321 |
| APP | 861 | CD86_ECD_V | 318 |
| CD19 | 813 | HSP90_C | 312 |
| FOLHD2 | 807 | CD3_E | 309 |
| TNF_SF11 | 783 | CD80_ECD_V | 303 |
| TACSTD | 729 | CD3_G | 282 |
| TNF_SF10 | 726 | CD3_D | 270 |
| CD33_ECD_full | 723 | IL5R_D1 | 243 |
| IL6R_DII_DIII | 720 | CA9_ECD_PG | 231 |
| IL5R_DII_DIII | 672 | PSCA | 225 |
| ANPT2_ECD1 | 663 | HSP90_CR | 201 |
| IL2B_FI_FII | 663 | IL2Ra_DI | 198 |
| IL2Ra_ECD_full | 660 | MS4AI_ECD2 | 186 |
| AMOT_ECD_2 | 642 | CD52_ECD | 111 |
| ICOSLIG_ECD | 633 | TNF_SF13_flap | 93 |
| CD80_ECD_full | 624 | CD247 | 90 |

**Table S3.** Target genes transferred from pAff8c vector to other expression vectors
