# Supplementary figures and images for: Automated Solid-Phase Subcloning Based on Beads Brought into Proximity by Magnetic Force
Source: PLoS One. 2012 May 18;7(5):e37429. doi: 10.1371/journal.pone.0037429 (PMC3356258; doi:10.1371/journal.pone.0037429)

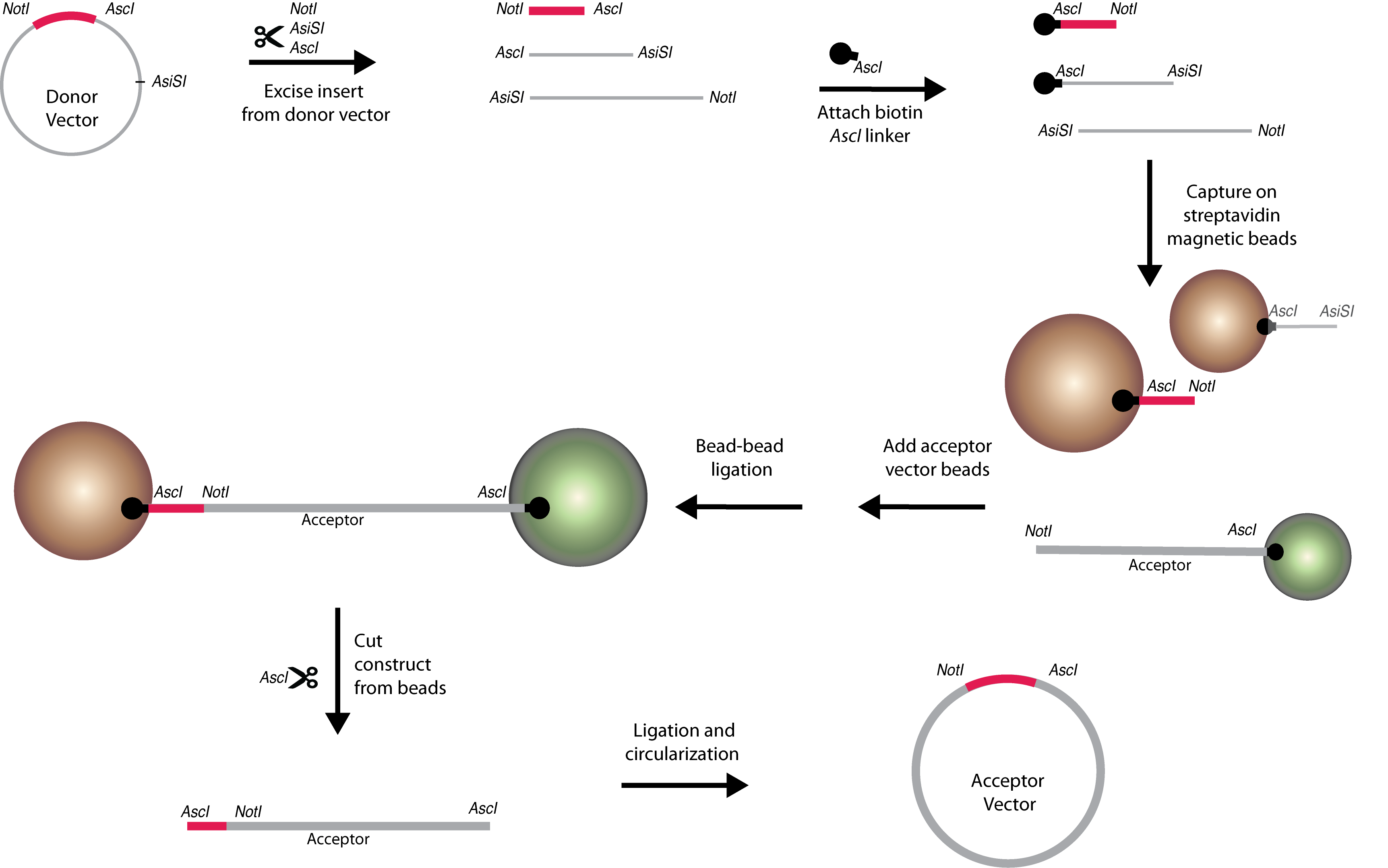

Supplement: Figure S1 — Detailed bead-based subcloning strategy. (TIF) [file pone.0037429.s004.tif]

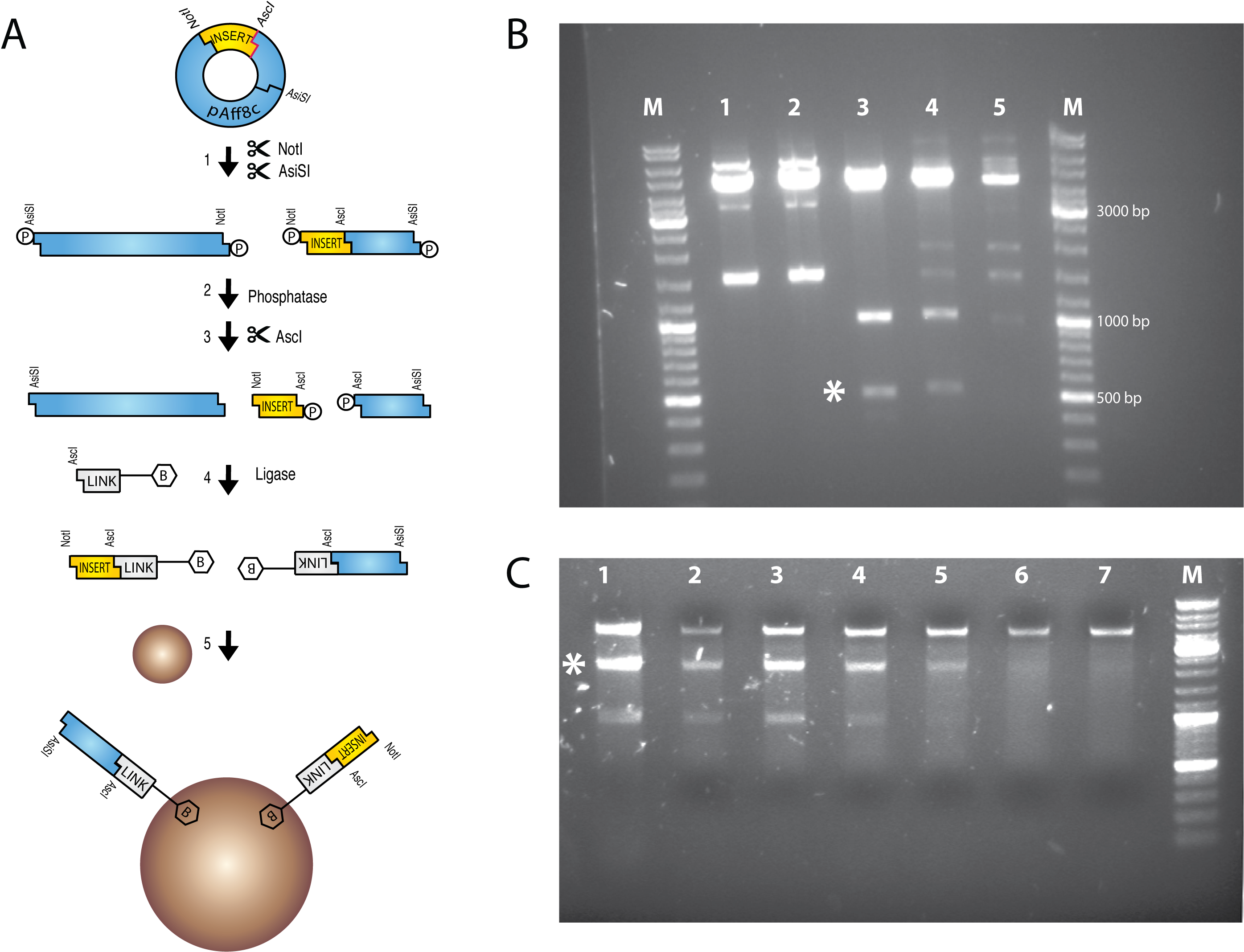

Supplement: Figure S3 — Detailed schematic of donor-bead construction and characterization. (TIF) [file pone.0037429.s006.tif]
