# Supplementary material for: Automated Solid-Phase Subcloning Based on Beads Brought into Proximity by Magnetic Force
Source: PLoS One. 2012 May 18;7(5):e37429. doi: 10.1371/journal.pone.0037429 (PMC3356258; doi:10.1371/journal.pone.0037429)
Supplement: Methods S1 — Preparation of acceptor plasmids. Preparation of acceptor beads from acceptor plasmids. Preparation of pAff8c library. Preparation of donor beads from pAff8c library. Automation of bead-to-bead protocol. Transfection of CHO-S and mammalian cell culture. (DOC) [file pone.0037429.s007.doc]

**SUPPLEMENTAL INFORMATION from**

Automated solid-phase subcloning based on beads brought into proximity by magnetic force

Elton P Hudson^1^, Andrej Nikoshkov^1^, Mathias Uhlen ^1,2,3^ and Johan Rockberg^1^

^1^School of Biotechnology, AlbaNova University Center, Royal Institute of Technology, Stockholm, Sweden, ^2^ Science for Life Laboratory, Royal Institute of Technology, Stockholm, Sweden, ^3^ The Novo Nordisk Foundation Center for Biosustainability, Technical University of Denmark, Denmark

**SUPPLEMENTAL INFORMATION**

*Supplemental Tables and Figures*

Table S1. Vectors used in this study

Table S2. Oligos used in this study

Table S3. Antigens in the pAff8c donor library

Figure S1. Detailed bead-based subcloning strategy

Figure S2. Size distribution of pAff8c donor inserts

Figure S3. Details of donor-bead construction

*Supplemental Methods S1*

Preparation of acceptor plasmids

Preparation of acceptor beads from acceptor plasmids

Preparation of pAff8c library

Preparation of donor beads from pAff8c library

Automation of bead-to-bead protocol

Mammalian cell culture

| **Table S1.** Vectors used in this study | | |
| --- | --- | --- |
| **Vector** | **Description** | **Size**  **(kb)** |
| pAff8c | *E. coli* intracellular vector. ”Donor” vector in bead-to-bead ligations | 5.6 |
| pHISZ | *E. coli* intracellular vector, used in fluorescent capping study | 2.8 |
| pSCEM2 | *S. carnosus* surface display vector | 7.8 |
| pPICZαCP | *P. pastoris* expression vector, modified from commercial pPICZαC | 3.8 |
| pLentiHAP | Mammalian expression vector, modified from commerical pLentiHAIII | 8.2 |

| **Table S2.** Oligos used in the construction of acceptor vectors and primers | | |
| --- | --- | --- |
| **Oligo** | **Description** | **Sequence** |
| LINK_F  LINK_R | Linking oligos. Contain AscI site and a 5’ biotin | 5’ CGCGCCTTCTGCAGGATCCTT 3’  3’ GGAAGACGTCCTAGGAA 5’ (biotin) |
| BamCAP_F  BamCAP_R | Fluorescent cap oligos for pHISZ. Contain BamHI site and a 5’AF488 | 5’GATCCACTGACTCGCATCCGATCGTG 3’  3’ GTGACTGAGCGTAGGCTAGCAC 5’ (AF488) |
| pPICZαC_ins | Insertion into EcoRI and XbaI of pPICZα-C to form pPICZα-CP. Contains NotI and AscI (italics). | 5’ GAATTCACATATGGGAAGCACGAGCTCCTCC*GCGGCCGC*TGCGAGC*GGCGCGCC*ACATCTAGA 3’ |
| pLenti_ins | Insertion into XmaI and XhoI of pLenti-HA to form pLenti-Hap. Contains NotI, AscI (italics), 6-His and Igk signal sequence (bold) | 5’ CCCGGGATGGAGACAGACACACTCCTGCT**ATGGGTACTGCTGCTCTGGGTTCCAGGTTCCACTGGTGAC**TCCGGGCATCACCATCACCATCACCATATGTCCATGAGCTCC*GCGGCCGC*TGGCTCT*GGCGCGCC*ACTCGAG 3’ |
| SAPA23 | pSCEM2 sequencing primer F | 5’ GGCTCCTAAAGAAAATACAACGGC 3’ |
| SAPA24 | pSCEM2 sequencing primer R | 5’ TGTTGAATTCTTTAAGGGCATCTGC 3’ |
| AOXF | pPICZαCp sequencing primer | 5’ GACTGGTTCCAATTGACAAGC 3’ |
| AOXR | pPICZαCp sequencing primer | 5’ GCAAATGGCATTCTGACATCCTCTTG 3’ |
| CMVF | pLentiHAp sequencing primer | 5’ CGCAAATGGGCGGTAGGCGTG 3’ |
| CMVR | pLentiHAp sequencing primer | 5’ TAGTCAGCCATGGGGCGGAGA 3’ |

**Table S3.** Target genes transferred from pAff8c vector to other expression vectors.

**Figure S1. Detailed bead-based subcloning strategy**. **(A)** An insert is excised from its donor vector, and capped with a biotin-containing oligo in a one-pot reaction. The insert is then attached to a streptavidin-coated bead and ligated to an immobilized acceptor vector, which has a compatible restriction site (here NotI). A non-productive fragment of the donor vector is also attached to bead, but cannot participate in further ligation steps due to a non-compatible restriction site (AsiSI). The DNA construct is released from bead via cutting at unique restriction site (here AscI).


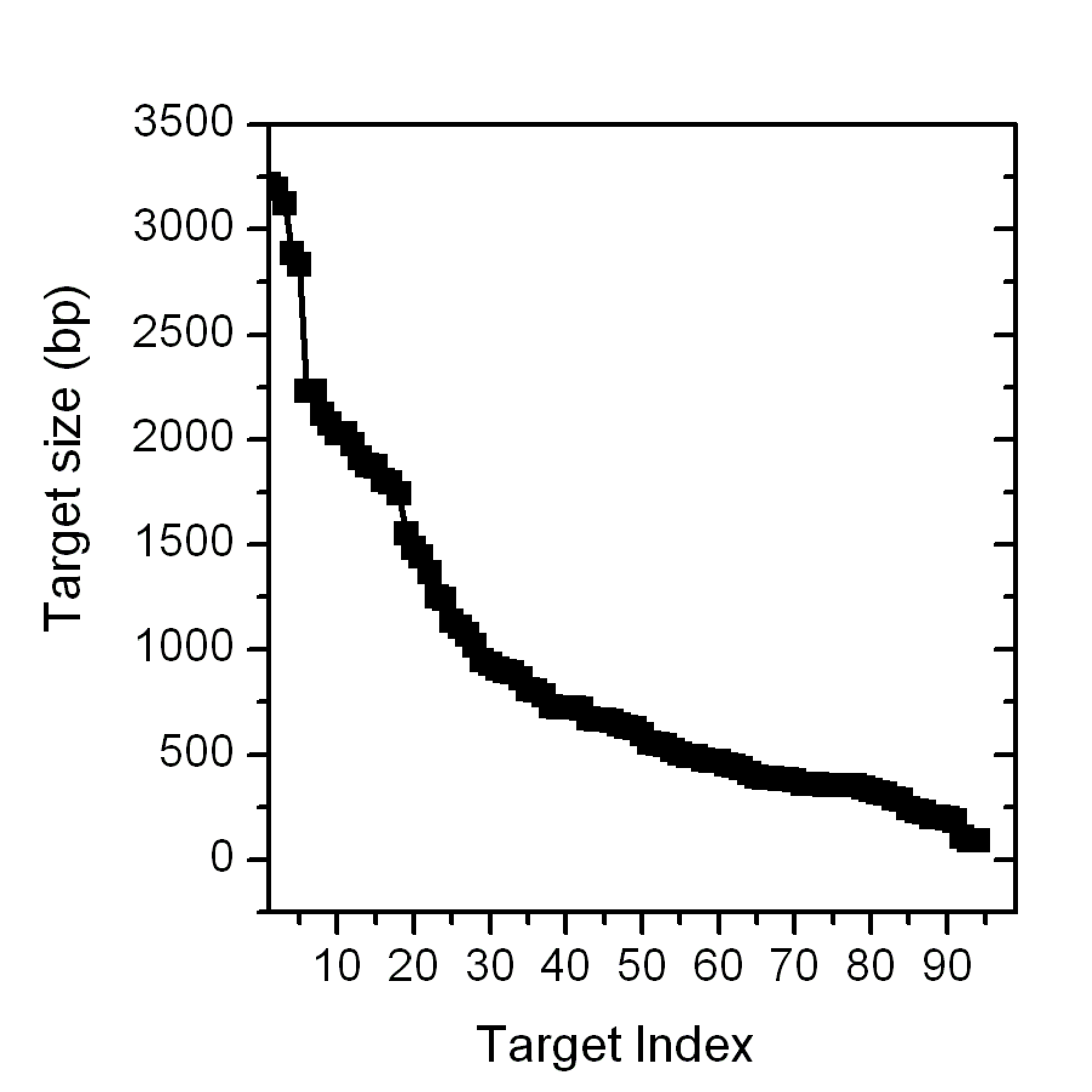


**Figure S2.** Size distribution of the 95 inserts listed in Table S3.

**Figure S3** Preparation of target gene for bead attachment during bead-bead subcloning. A) Schematic of the sequential steps for excision of a target gene from its donor vector, attachment of a biotinylated oligo, and capture by M270 beads. The steps are performed in one pot, with washing of the bead after magnetic capture. The bead is then used in bead-bead ligation with an acceptor-vector bead. B) Target gene mixture at each stage 1-5 depicted in 1A. The concentration was 10 ng DNA/μL. The donor vector is pAff8c. The target gene (here TNF 500 bp) is marked with a star. At stage 5 the target gene is removed from solution during bead capture. A second DNA fragment is also attached to the bead, but cannot participate in bead-bead ligation due to its incompatible restriction site. C) Titration of the mixture in step 5 of 1A with varying amounts of bead. The insert (here EGFR 2000 bp) is marked with a star. From left to right: 1-no beads, 2- beads at 0.05 μg/μL, 3-0.1 μg/μL, 4-0.5 μg/μL, 5- beads at 1 μg/μL, 6- beads at 5 μg/μL, 7-beads at 10 μg/μL. The beads capture all of the biotinylated insert at 0.5 μg/μL. This amount was chosen as optimal to maximize insert attachment. Additionally, no vector DNA is attached to the beads under these conditions.

MATERIALS AND METHODS

*Supplemental Methods S1*

Preparation of acceptor plasmids

Preparation of acceptor beads from acceptor plamids

Preparation of pAff8c library

Preparation of donor beads from pAff8c library

Automation of bead-to-bead protocol

*Construction of acceptor plasmids*

The *Pichia pastoris* expression vector pPICZαCp was formed by addition of *NotI* and *AscI* restriction sites into the MCS of pPICZαC (Invitrogen). The coding sequence is given in Table S2. The mammalian expression vector pLentiHAp was formed through addition of an Ig-κ signal peptide, an N-terminal *His6* tag and NotI and AscI restriction sites into the MCS of pLenti-HA plasmid (Applied Biological Materials, Canada). The *Staphylococcus carnosus* expression vector pSCEM2 was described previously (1). The *E. coli* expression vector pAff8c was described previously(2). The vector pHISZ was used for the fluorescent capping study, which codes for a S.aureus Protein A Z-domain fused to eGFP.

*Preparation of pSCEM2, pPICZ*α*P and pLentiHAP acceptor beads*

Acceptor plasmids were linearized with NotI and AscI (10 μg DNA, 100 μL reaction; 37 C 2 h, 80 C 5 min). The plasmid DNA was purified via PCR cleanup kit (Qiagen). A double-stranded biotinylated linker containing a complementary AscI restriction site was attached to the *AscI* end via T4 DNA ligase (10 μg plasmid, 125 pmol linker). The mixture was treated again with NotI (1 hr 37 C), purified via PCR cleanup kit, and dissolved in 500 μL buffer EB.

5 mg of M270 beads were washed 3 times and re-suspended in 500 μL of 2X BW buffer, which was prepared according to the supplier’s protocol with the addition of 10% v/v PEG4000. DNA solution (10 μg) and M270 beads (5 mg) were combined (1 mL) and incubated with constant shaking for 2 h at 25 C. The beads were removed from solution via magnetic separation, washed 3 times with 1X BW buffer and re-suspended in 1 mL 1X BW. The amount of plasmid DNA attached to the beads was determined by incubating the DNA-coated beads with *AscI* at 37 C for 2h and quantification of released DNA on agarose gel.

*Preparation of pHISZ acceptor beads*

The pHISZ vector was used as a PCR template and a biotinylated primer was used in the reaction. The PCR product was attached to beads as described above. The beads were then incubated with BamHI (37 C 1 hr) to make a BamHI site available for the ligation of the fluorescent cap. As a positive control for the fluorescent capping study, a PCR was performed with one biotinylated primer and one fluorescent (AF488) primer. The PCR product was then attached to beads as described above.

*Construction of the pAff8c clone library*

The pAff8c clones were used as the “donor” vectors in solution-to-bead and bead-to-bead ligations. A 95-member pAff8c clone library was constructed, consisting of the partial ectodomains of 65 cancer antigens. The NovoNordisk Foundation Center for Protein Research at the University of Copenhagen, Denmark kindly provided clones containing the full-length genes in various shuttle vectors. Gene-specific PCR primers containing NotI and AscI sites were designed for amplification of the antigen gene fragments. The NotI-containing forward primer contained 5’-biotin, which allowed for purification via M270 streptavidin-coated beads and subsequent mangetic separation of the PCR product. PCR purification was performed in 96-well format on a Magnatrix 8000 robotic liquids handler: PCR product was incubated with M270 beads, which were then magnetically separated from solution. Bead-bound PCR product was treated with AscI at 37 C, washed twice, and released from bead via treatment with NotI at 37 C. The purified amplicons were then ligated into a NotI-AscI-digested pAff8c plasmid and used for bacterial transformation in 96-well format. All clones were sequenced-verified by Sanger sequencing. pAff8c vectors were purified from cell-culture with a Montage 96-well miniprep kit (Millipore).

*Preparation of donor beads for bead-to-bead ligation*

Preparation of donor beads involved the excision of the target DNA from vector pAff8c, attachment of a biotinylated linker to the target sequence, and subsequent capture by M270 beads.

50 ng of pAff8c was incubated with NotI, AsiSI and FastAP phosphatase (50 μL reaction volume; 0.5 μL of each enzyme, 37 C 30 min then 80 C 5 min). This three-enzyme mixture was prepared as a stock solution and stored in 40% glycerol at -20 C. AscI (0.5 μL) was then added and the reaction incubated at 37 C 20 min, then 65 C 20 min. The free AscI ends were then capped by addition of the biotinylated linking oligo LINK(1.5 pmol), which was ligated in the presence of PEG4000 2% v/v at 22 C 30 min. A schematic of the donor bead preparation is shown in Figure S1A. Optimization of the LINK addition step is in Figure S1B.

Biotinylated DNA was captured with addition of M270 beads (50 μL at 0.5 μg/μL) and shaking at 22 C 45 min. DNA-coated beads were removed from solution via magnetic separation and washed twice with 1X BW solution and suspended in Tris buffer pH 8. Typical DNA attachment was at ~1 ng DNA/μg beads. Optimization of the M270 beads incubation step is shown in Figure S1C.

*Automation of the subcloning procedure*

The subcloning protocol was programmed onto the Magnatrix 8000 robot using the manufacturer’s software. The pAff8c-donor library was used in 96-well format. Enzymes, M270 beads and acceptor beads were prepared as reagents and stored at 4 C during the procedure.

Reagent List (per reaction)

I – 0.2 μL each NotI, AsiSI, FastAP in 10 μL 1X FastDigest buffer

II- 0.2 μL AscI in 10 μL 1X FastDigest buffer

III- 0.4 pmol biotinylated AscI-LINK oligo in Tris pH 8, 20% v/v PEG4000

IV- 0.5 μL T4 DNA ligase in 10 μL 1X T4 DNA ligase buffer

V- 5 μL naked M270 (0.1 μg/μL) beads in 2X BW buffer

VI- Acceptor beads: 2 μL of pLentiPA loaded at 0.5 ng DNA/μg beads in EB buffer.

Procedure; added reagents are in (). Donor vector was 100 ng in 20 μL EB

1) Digestion of pAff8c. Add (I) 30 min 37 C ; denaturation 80 C 5min

2) Digestion of pAff8c. Add (II) 15 min 37 C; denaturation 65 C 15 min

3) Attachment of biotynlated *AscI*-LINK oligo Add (III, IV); 22 C 15 min

4) Attachment of biotinylated DNA to M270 beads. Add (V); 22 C 30 min with pipette stirring every 5 min

5) Washing of DNA-loaded beads via magnetic separation, elute in Tris pH 8

6) Ligation of donor- and acceptor beads Add (VI, IV); 22 C 1 hr with magnet holding; pipette stirring every 15 min.

7) Detachment of DNA from beads. Add (II); 37 C 30 min with pipette stirring every 5 min; denaturation 65 C 15 min.

8) Removal of beads from solution via magnetic separation and discard.

9) Circularization of DNA. Add (IV); 22 C 10 min; hold 4 C

The completed vectors were used for bacterial transformation in 96-well format. Resulting colonies were PCR-screened using pLentipA-specific primers CMVF and CMVR.

*Mammalian cell culture and protein expression*

The HEK293T (293tsA1609neo) cell line expressing the SV40 large T antigen

(ATCC) and CHO-S cells (Invitrogen) were cultivated in Dulbecco's modified Eagle's medium (DMEM) (Sigma) supplemented with 10% heat inactivated bovine calf serum (Sigma) and 100 IU/ml penicillin and streptomycin. Three hours prior transfection of HEK293T cells DMEM growing media was changed to Iscove's modified Dulbecco medium (Sigma) supplemented with 10% heat inactivated bovine calf serum (Sigma) and 100 IU/ml penicillin and streptomycin. For studying recombinant protein production transformed CHO-s cells were cultivated in Ex-cell CHO serum-free media (SAFC biosciences).

For lentiviral particles (LVP) production 700 000 HEK 293T cells were plated on a 6cm Falcon culture plates coated with poly-L-Lysine (Sigma). After 18 hours the 293T cells were transfected via calcium precipitation (3) with 8 μg lentiviral backbone vector, 6 μg packaging construct, and 2.4 μg plasmid encoding the VSV-G protein. After 18 hours medium was exchanged and stabilized by 10 mM HEPES for lentivirus accumulation and further CHO-S cells transfection. Briefly, 18-24 hours after beginning virus collection media containing LVP was supplemented with 8 μg/ml of polybrene and applied to 150000 CHO-S cells seeded on 6 cm culture plates 18 hours before adding LVP suspension. 24 hours later LVP containing media was replaced with fresh DMEM media, CHO cells were further incubated for 48 hours and passed into growth media containing 10 μg/ml of puromycin for selection of stable transformants. 30 to 50% of LVP infected CHO cells recovered in selective media and were used for recombinant protein production. After reaching confluence selected stably transformed CHO-S cells were passed into puromycin containing DMEM growth media. After reaching 70-80% confluence growth media was replaced with CHO minimal media without puromycin and cells were incubated for one week. Cells were sonicated (Sonics VC750; 1 min, pulse 1s, power 25 %) in lysis buffer (20 mM Tris-HCl pH 6.8, 150 mM NaCl, 1% Triton-X-100) and lysates were analyzed by Western blot.

1. Rockberg,J., Lofblom,J., Hjelm,B., Uhlen,M. and Stahl,S. (2008) Epitope mapping of antibodies using bacterial surface display. *Nature Methods*, **5**, 1039-1045.

2. Larsson,M., Gräslund,S., Yuan,L., Brundell,E., Uhlén,M., Höög,C. and Ståhl,S. (2000) High-throughput protein expression of cDNA products as a tool in functional genomics. *Journal of biotechnology*, **80**, 143-57.

3. Jordan,M., Schallhorn, a and Wurm,F.M. (1996) Transfecting mammalian cells: optimization of critical parameters affecting calcium-phosphate precipitate formation. *Nucleic acids research*, **24**, 596-601.
